# Histopathological assessment of radial artery calcification in patients with end-stage kidney disease

**DOI:** 10.1080/0886022X.2021.1889600

**Published:** 2021-03-09

**Authors:** Zhenwei Chen, Youjian Zhou, Tiecheng Yang

**Affiliations:** a Department of Nephrology, The Eighth Affiliated Hospital of Sun Yat-sen University, Shenzhen, Guangdong, PR China; b Department of Pathology, The Eighth Affiliated Hospital of Sun Yat-sen University, Shenzhen, Guangdong, PR China

**Keywords:** Dialysis, diabetes mellitus, vascular calcification, chronic kidney disease

## Abstract

**Background:**

A comprehensive understanding of vascular calcification pathology is significant for the development of cardiovascular disease therapy in high-risk populations. This cross-sectional study aimed to evaluate the prevalence and characteristics of radial artery calcification (RAC) and to identify the factors that are associated with RAC in end-stage kidney disease (ESKD).

**Methods:**

Detailed medical histories of 180 patients with ESKD were recorded. Fragments of the radial artery obtained during the creation of arteriovenous fistula for hemodialysis access were stained with alizarin red S.

**Results:**

Calcification was localized in the arterial media layer. The prevalence of positive calcification staining in the radial arteries was 21.1% (*n* = 38). Patients with RAC had a higher glycated hemoglobin level (*p* < 0.01), higher prevalence of dialysis duration >5 years (*p* = 0.022), and diabetes mellitus (*p* < 0.01) than those without RAC. Multiple logistic regression models showed dialysis duration >5 years (odds ratio [OR], 9.864; 95% confidence interval [CI], 2.666–36.502; *p* < 0.01) and diabetes mellitus (OR, 12.689; 95% CI, 2.796–34.597; *p* < 0.01) were independent risk factors for RAC in patients with ESKD. Patients with dialysis duration >5 years had a higher prevalence of RAC (*p* = 0.012) than those with dialysis duration ≤5 years. Patients with diabetes mellitus had a higher prevalence of RAC (*p* < 0.01) than those without diabetes mellitus. Patients with diabetes mellitus ≥15 years had a higher prevalence of RAC (*p* = 0.042) than those with diabetes mellitus <15 years. Radial artery calcification level showed a significantly positive correlation with dialysis duration (*p* < 0.05), diabetes mellitus duration (*p* < 0.01), HbA1c level (*p* < 0.01) and Calcium level (*p* < 0.01).

**Conclusions:**

In patients with ESKD, dialysis duration >5 years and diabetes predict RAC. Thus, the combination of prolonged dialysis and hyperglycemic conditions exerts a synergistic effect on RAC.

## Introduction

Cardiovascular disease is the leading cause of mortality in patients with end-stage kidney disease (ESKD), and vascular calcification (VC) is an independent predictor of vascular morbidity and mortality [1,2]. VC is defined as the abnormal deposition of calcium phosphate salts in vascular tissue, including the valves, blood vessels, and heart. VC can occur in the intima and media of the vasculature. Arterial intimal calcification (AIC), which is related to inflammation, appears mainly in the early stage of chronic kidney disease (CKD), whereas arterial medial calcification (AMC), also known as Monckeberg’s sclerosis, mainly occurs in the advanced stage of CKD [3]. A previous study demonstrated that AMC is an active cell-mediated process associated with mineral metabolism disturbance [4], and the vascular smooth muscle cell (VSMC) phenotypic changes result in the promotion of upregulation of osteogenic programs [5].

In general, VC can be detected using plain radiographs, two-dimensional ultrasonography, electron-beam computed tomography, and multi-detector computed tomography [6,7]. However, there are some limitations in these noninvasive imaging techniques when used in patients with VC. The gold standard for diagnosing VC is the histological examination of arterial specimens, which is the most accurate histological assessment to locate calcification and determine severity.

A comprehensive understanding of VC pathology is important for the development of cardiovascular disease therapy in high-risk populations. In our study, we used radial arterial wall specimens obtained during arteriovenous fistula (AVF) creation for hemodialysis (HD) access. The aims of this study were to evaluate the prevalence and characteristics of RAC and to identify the factors associated with the presence of RAC in patients with ESKD.

## Materials and methods

### Ethics statements

All procedures performed in our study involving human participants were in accordance with the principles of the Declaration of Helsinki and in compliance with the International Conference on Harmonization/Good Clinical Practice regulations. According to the Chinese Law, the study was approved by The Eighth Affiliated Hospital of Sun Yat-sen University’s ethics committee (number KY2016-002). Written consent was provided by all participants.

### Patients

Adult patients with ESKD undergoing AVF surgery between July 2015 and September 2017 at The Eighth Affiliated Hospital of Sun Yat-sen University were invited to participate in this cross-sectional study. Patients with a history of peritoneal dialysis and history of kidney transplantation, parathyroidectomy, and life-threatening comorbid conditions, such as malignancy and active infection, occurring within the past 3 months were excluded. The 180 specimens were obtained by two surgeons from the same medical center during the process of AVF creation.

Patients’ detailed medical histories, including age, sex, diabetes mellitus, hypertension, past or current smoking, duration of dialysis, medications, serum biochemical data, and blood cell counts, were recorded. Patients' symptoms, signs, and serum indexes were comprehensively monitored to detect any confounding condition that may affect VC. The data collector was blinded to patients' clinical and laboratory data.

### Laboratory measurements

Fasting blood samples were obtained prior to AVF surgery, centrifuged within 1 h after collection, and then immediately sent to the central laboratory for analysis. The measured biochemical serum parameters included corrected serum calcium, phosphorous, albumin, creatinine, blood urea nitrogen, alkaline phosphatase, total cholesterol, low-density lipoprotein (LDL), and triglyceride levels, using direct chemiluminescent immunoassay (Beckman Coulter UniCel DxC 800 Synchron, Brea, CA, USA). Serum intact parathyroid hormone (iPTH) levels were measured using a Centaur Intact iPTH assay (Siemens Healthcare Diagnostics, Inc., Tarrytown, NY, USA). High-sensitivity C-reactive protein levels were determined by immunoturbidimetry (Olympus, Rungis, France).

### Histology

One piece (0.3–1 cm long) of the radial artery was collected during AVF surgery, and it was immediately fixed in 4% neutral formaldehyde. After fixation, the radial arteries were embedded in paraffin and cut with a rotating microtome at 2-μm thickness. The 2-μm-thick sections were stained with alizarin red S. Positive results indicated by an orange-red color were shown under the light microscope. An experienced pathologist evaluated the stained sections and examined them using an Olympus microscope (Olympus, Tokyo, Japan) in bright-field mode. The images were recorded using an Olympus DP-71 digital CCD camera, which was under software control, and medial calcification was graded by the pathologist on a semi-quantitative scale (0 = none; 1 = mild; 2 = moderate; and 3 = severe).

### Statistical analysis

Continuous variables are presented as medians from 25th to 75th percentiles and categorical data as proportions. Chi-square analysis or the Fisher exact test was used to compare categorical variables, as appropriate. Normally distributed continuous variables were analyzed using the Student t-test or analysis of variance, whereas non-normally distributed continuous variables were analyzed using the non-parametric Kruskal–Wallis test. Correlation analysis was performed using Spearman’s rank correlation. Multivariate statistical analysis (logistic regression, enter method) was used to evaluate possible RAC confounders, including diabetes mellitus, dialysis duration >5 years, and glycated hemoglobin (HbA1c). A two-sided *p* < 0.05 was considered statistically significant. All analyses were performed using SPSS version 22.0 (IBM Corp., Armonk, NY, USA).

## Results

### Baseline patient characteristics

The baseline characteristics of the 180 patients are presented in Table 1. All patients were Chinese, with a median age of 49.5 (range, 41.0–64.8) years, and 60% were men, while 34.4% had diabetes. Of these, 160 patients (88.9%) had already initiated dialysis therapy before AVF creation. The causes of ESKD were chronic glomerulonephritis (*n* = 83), diabetic nephropathy (*n* = 50), angiosclerosis and hypertensive nephropathy (*n* = 19), obstructive interstitial nephropathy (*n* = 8), polycystic kidney disease (*n* = 8), lupus nephritis (*n* = 4), and others (*n* = 8).

**Table 1.** Differences in clinical parameters between patients with and without VC.

Factor	All patients (*n* = 180)	No VC (*n* = 142)	VC (*n* = 38)	*p*
Age (years)	49.5 (41.0–64.8)	50.0 (40.8–67.3)	49.0 (40.8–64.0)	0.596
Male sex (%)	108 (60.0)	83 (58.5)	25 (65.8)	0.412
Smoking habit (%)	39 (21.7)	30 (21.1)	9 (23.7)	0.734
Diabetes mellitus (%)	62 (34.4)	36 (25.4)	26 (68.4)	0.001*
Dialysis duratio*n* > 5 years (%)	32 (17.8)	20 (14.1)	12 (31.6)	0.022*
Systolic BP (mm Hg)	160.0 (143.0–173.8)	159.0 (142.0–171.3)	164.0 (151.0–170.3)	0.243
Diastolic BP (mm Hg)	89.0 (80.0–100.8)	90.5 (80.0–101.0)	85.0 (80.0–97.0)	0.152
Hemoglobin level (g/L)	96.5 (81.0–114.0)	97.0 (81.0–114.0)	96.5 (80.8–109.5)	0.617
Triglyceride level (mmol/L)	1.28 (0.97–1.91)	1.26 (0.98–1.88)	1.43 (0.94–2.09)	0.522
Cholesterol level (mmol/L)	4.27 (3.64–4.88)	4.30 (3.64–4.96)	4.10 (3.63–4.77)	0.774
LDL-cholesterol level (mmol/L)	2.30 (1.90–2.80)	2.25 (1.90–2.90)	2.37 (1.80–2.80)	0.726
Creatinine level (μmol/L)	832.0 (615.5–1158.5)	843.0 (614.8–1159.8)	805.5 (666.0–1180.8)	0.593
BUN level (mmol/L)	20.6 (15.5–25.6)	20.8 (15.5–25.5)	19.4 (15.3–26.2)	0.971
Albumin level (g/L)	33.3 (30.5–36.8)	33.6 (30.6–37.2)	32.9 (28.0–36.0)	0.073
hs-CRP level (mg/L)	4.0 (1.0–6.0)	4.0 (1.0–6.0)	4.0 (2.0–7.3)	0.101
HbA1c level (%)	5.7 (5.1–6.2)	5.6 (5.1–6.1)	6.2 (5.4–8.1)	0.009*
Calcium carbonate supplements (%)	148 (82.2)	115 (81.0)	33 (86.8)	0.402
Calcitriol supplements (%)	163 (90.6)	128 (90.1)	35 (92.1)	0.713
iPTH level (pg/mL)	235.7 (143.7–446.1)	235.7 (146.4–423.0)	221.6 (131.7–630.4)	0.920
Calcium level (mmol/L)	2.41 (2.22–2.53)	2.39 (2.21–2.48)	2.50 (2.32–2.63)	0.107
Phosphate level (mmol/L)	1.94 (1.53–2.46)	1.94 (1.56–2.41)	1.92 (1.50–2.50)	0.820
Calcium-phosphorus product level (mg^2^/dL^2^)	56.36 (43.83–72.73)	55.89 (43.77–70.02)	59.05 (44.13–70.11)	0.309
Homocysteine level (μmol/L)	20.0 (15.8–26.1)	20.3 (16.1–25.3)	18.7 (12.1–26.5)	0.262

VC: vascular calcification; BP: blood pressure; HbA1C: glycated hemoglobin A1C; hs-CRP: high-sensitivity C-reactive protein; BUN: blood urea nitrogen; iPTH: intact parathyroid hormone; LDL-C: low-density lipoprotein cholesterol.

*p < 0.05.

### Histological findings

**Figure 1.**
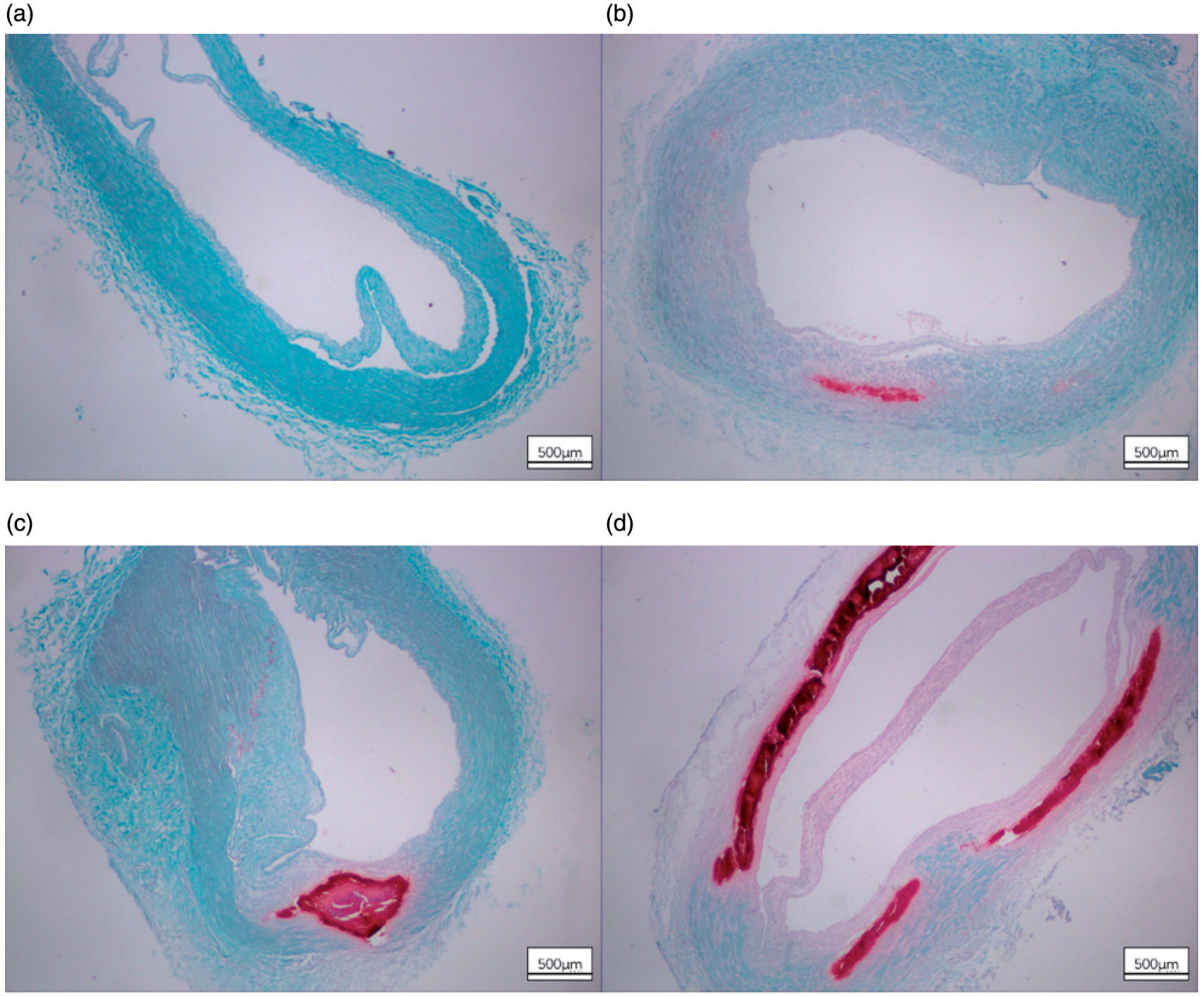
Consecutive sections of radial arteries stained by alizarin red S indicate the calcification of varying grades. Intimal thickening and no mineral content (a). Small calcified section in the medial layer (b). Large calcified sections localized on the medial layer (c). Serial sections of the entire arterial wall sample demonstrating advanced calcification (d).

In all included patients with ESKD, the calcification stained by alizarin red S (Figure 1(a–d)) in the arterial medial layer was found in 21.1% (*n* = 38). Overall, 148 patients did not have calcification, seven had minor calcification, 14 had moderate calcification, and 17 had severe calcification. Basophilic deposits were visible on the arterial walls in cases of advanced calcification that presented with varying growth. However, the stained arterial specimens did not show any intimal layer calcification. Intimal thickening was found in most arterial specimens.

### Correlations between radial artery calcification and clinical parameters

Table 1 summarizes the clinical and biochemical parametric differences between the groups with and without RAC as assessed by alizarin red S. Patients with RAC had a higher HbA1c level (6.2% versus [vs.] 5.6%; *p* < 0.01), higher prevalence of dialysis duration >5 years (31.6% vs. 14.1%; *p* = 0.022), and diabetes mellitus (68.4% vs. 25.4%; *p* < 0.01) than those without RAC. In traditional VC risk factors, the two groups did not differ from having a statistically significant difference in age, sex, smoking habits, hypertension, and dyslipidemia (all, *p* > 0.05). In nontraditional risk factors, they did not statistically significant differ in high-sensitivity C-reactive protein, calcium, phosphate, and homocysteine levels (*p* > 0.05). Multivariate statistical analysis (logistic regression, enter method) (Table 2) showed that dialysis duration >5 years (odds ratio [OR], 9.864; 95% confidence interval [CI], 2.666–36.502; *p* < 0.01), and diabetes mellitus (OR, 12.689; 95% CI, 2.796–34.597; *p* < 0.01) were independent risk factors for RAC.

**Table 2.** Multiple logistic regression models showing the associations of the selected variables with RAC.

Variable	Simple Model		Model 1^a^		Model 2^b^	
	OR (95% CI)	*p*	OR (95% CI)	*p*	OR (95% CI)	*p*
Dialysis duratio*n* > 5 years (%)	2.815 (1.226–6.466)	0.015*	10.603 (2.904–38.722)	0.001*	15.899 (3.548–71.236)	0.001*
Diabetes mellitus (%)	6.380 (2.920–13.939)	0.001*	8.758 (2.547–30.117)	0.001*	12.519 (2.811–55.757)	0.001*
HbA1c level (%)	1.703 (1.286–2.255)	0.001*	1.341 (0.956–1.883)	0.090	1.455 (0.973–2.175)	0.068
Calcium level (mmol/L)	1.969 (1.256–3.089)	0.003*	1.459 (0.888–2.397)	0.136	3.753 (0.593–23.756)	0.160
Albumin level (g/L)	0.938 (0.883–0.996)	0.036*	0.954 (0.882–1.031)	0.234	0.948 (0.864–1.040)	0.257
Age (years)	1.007 (0.982–1.031)	0.600	–	–	0.970 (0.930–1.012)	0.161
Smoking habit (%)	1.159 (0.495–2.709)	0.734	–	–	0.752 (0.227–2.489)	0.640
Triglyceride level (mmol/L)	1.164 (0.816–1.661)	0.402	–	–	0.822 (0.460–1.469)	0.509
Cholesterol level (mmol/L)	0.952 (0.683–1.328)	0.773	–	–	0.821 (0.317–1.469)	0.685
LDL-cholesterol level (mmol/L)	0.944 (0.592–1.507)	0.809	–	–	1.309 (0.398–4.298)	0.658
Homocysteine level (μmol/L)	0.988 (0.956–1.021)	0.477	–	–	1.001 (0.965–1.039)	0.950
Calcium carbonate supplements (%)	1.550 (0.553–4.339)	0.404	–	–	0.908 (0.184–4.473)	0.906
Calcitriol supplements (%)	1.276 (0.347–4.691)	0.714	–	–	1.168 (0.182–7.505)	0.870
iPTH level (pg/mL)	1.000 (0.999–1.001)	0.639	–	–	1.000 (0.999–1.001)	0.466
Phosphate level (mmol/L)	1.037 (0.885–1.215)	0.653	–	–	3.598 (0.288–44.999)	0.321
Calcium-phosphorus product level (mg^2^/dL^2^)	1.009 (0.994–1.025)	0.240			0.874 (0.671–1.138)	0.317
Systolic BP (mm Hg)	1.007 (0.993–1.022)	0.332			1.000 (0.974–1.027)	0.978
Diastolic BP (mm Hg)	0.982 (0.957–1.007)	0.152			1.004 (0.956–1.053)	0.883
Male sex (%)	1.367 (0.647–2.890)	0.413			1.371 (0.454–4.146)	0.576
Hemoglobin level (g/L)	0.996 (0.980–1.012)	0.615				
Creatinine level (μmol/L)	1.000 (0.999–1.001)	0.818				
BUN level (mmol/L)	1.003 (0.963–1.045)	0.888				
hs-CRP level (mg/L)	1.027 (0.956–1.102)	0.465				

RAC: radial artery calcification; BP: blood pressure; HbA1C: glycated hemoglobin A1C; hs-CRP: high-sensitivity C-reactive protein; BUN: blood urea nitrogen; iPTH: intact parathyroid hormone; LDL-C: low-density lipoprotein cholesterol; OR: odds ratio; CI: confidence interval.

Odds ratios for positive staining are presented.

^a^ Adjusted for diabetes mellitus, HbA1c, and dialysis duration >5 years.

^b^ Adjusted for diabetes mellitus, HbA1c, and dialysis duration >5 years, age, smoking habit, triglyceride level, cholesterol level, LDL-cholesterol level, hs-CRP level, calcium supplements, vitamin D supplements, iPTH level, calcium level, phosphate level, and calcium-phosphorus product level.

*p < 0.05.

### Correlations between radial artery calcification and dialysis vintage

The left side of Table 3 shows the differences in clinical and laboratory results between patients undergoing predialysis and dialysis. Compare to patients with predialysis, those with dialysis had higher levels of hemoglobin (median, 99 vs. 82 g/L; *p* < 0.01), hs-CRP (median, 4 vs. 2 mg/L; *p* < 0.01), and calcium (median, 2.42 vs. 2.24 mmol/L; *p* < 0.01) and lower levels of BUN (median, 19.4 vs. 24.6 mmol/L; *p* = 0.023). The right side of Table 3 compares the clinical and laboratory results between patients with dialysis duration ≤5 years and dialysis duration >5 years. We discovered that if dialysis duration was >5 years, there were a higher prevalence of RAC (37.5% vs. 17.6%; *p* = 0.012); higher levels of hemoglobin (median, 113 vs. 94 g/L; *p* < 0.01), creatinine (median, 1185 vs. 794 μmol/L; *p* < 0.01), albumin (median, 35.7 vs. 33.0 g/L; *p* < 0.01), iPTH (median, 243.6 vs. 170 pg/mL; *p* < 0.01), calcium (median, 2.48 vs. 2.38 mmol/L; *p* < 0.01), phosphate (median, 2.49 vs. 1.85 mmol/L; *p* < 0.01), calcium-phosphorus product (median, 75 vs. 52.15 mg^2^/dL^2^; *p* < 0.01); and lower prevalence of diabetes mellitus (12.5% vs. 39.2%; *p* < 0.01). Moreover, the proportion of patients treated with calcium carbonate (96.9% vs. 79.1%; *p* = 0.019) was higher in the dialysis duration >5 years group than in the dialysis duration ≤5 years group.

**Table 3.** Correlations between dialysis duration and clinical and laboratory variables.

Factor	Predialysis (*n* = 20)	Dialysis (*n* = 160)	*p*	Dialysis duratio*n* ≤ 5 years (*n* = 148)	Dialysis duratio*n* > 5 years (*n* = 32)	*p*
Age (years)	47.0 (41.3–66.3)	50.0 (40.3–64.8)	0.668	51.0 (41.0–67.0)	47.0 (40.3–55.3)	0.057
Male sex (%)	9 (45.0)	99 (61.9)	0.146	85 (57.4)	23 (71.9)	0.130
Smoking habit (%)	3 (15.0)	36 (22.5)	0.573	31 (20.9)	8 (25)	0.614
Diabetes mellitus (%)	8 (40.0)	54 (33.8)	0.579	58 (39.2)	4 (12.5)	0.004*
RAC (%)	3 (15.0)	35 (21.9)	0.575	26 (17.6)	12 (37.5)	0.012*
Systolic BP (mm Hg)	164.0 (144.5–168.8)	160.0 (143.0–171.8)	0.812	160.0 (144.0–171.8)	158.5 (135.3–168.0)	0.145
Diastolic BP (mm Hg)	89.0 (77.8–99.3)	89.5 (80.0–101.0)	0.374	89.0 (80.0–100.0)	91.5 (81.5–102.5)	0.557
Hemoglobin level (g/L)	82.0 (71.3–93.0)	99.0 (82.3–116.0)	0.001*	94.0 (78.3–110.5)	113.0 (93.3–127.0)	0.001*
Triglyceride level (mmol/L)	1.26 (0.98–1.88)	1.28 (0.95–1.95)	0.549	1.25 (0.98–1.88)	1.48 (0.99–2.26)	0.102
Cholesterol level (mmol/L)	4.33 (3.92–5.13)	4.26 (3.61–4.83)	0.419	4.27 (3.62–4.83)	4.23 (3.72–5.11)	0.743
LDL-cholesterol level (mmol/L)	2.30 (1.90–2.60)	2.30 (1.80–2.88)	0.493	2.20 (1.90–2.88)	2.38 (1.83–2.75)	0.976
Creatinine level (μmol/L)	843.0 (614.8–1159.8)	805.5 (666.0–1180.8)	0.385	794.0 (589.0–1010.0)	1185.0 (951.0–1352.3)	0.001*
BUN level (mmol/L)	24.6 (19.3–31.5)	19.4 (15.3–26.2)	0.023*	20.2 (15.2–25.3)	21.6 (17.3–27.3)	0.273
Albumin level (g/L)	32.6 (30.8–35.2)	33.6 (30.4–37.3)	0.445	33.0 (30.0–36.0)	35.7 (32.4–38.7)	0.003*
hs-CRP level (mg/L)	2.5 (1.0–3.0)	4.0 (2.0–7.0)	0.008*	3.0 (1.0–6.0)	5.0 (2.3–10.0)	0.052
HbA1C level (%)	5.5 (4.9–5.6)	5.8 (5.1–6.3)	0.089	5.7 (5.1–6.3)	5.6 (4.9–6.0)	0.122
Calcium carbonate supplements (%)	14 (70.0)	134 (83.8)	0.129	117 (79.1)	31 (96.9)	0.019*
Calcitriol supplements (%)	16 (80.0)	147 (91.9)	0.102	132 (89.2)	31 (96.9)	0.315
iPTH level (pg/mL)	170.0 (98.4–290.0)	243.6 (150.0–458.9)	0.110	170.0 (98.4–290.0)	243.6 (150.0–458.9)	0.001*
Calcium level (mmol/L)	2.24 (2.06–2.42)	2.42 (2.23–2.55)	0.004*	2.38 (2.21–2.49)	2.48 (2.40–2.67)	0.001*
Phosphate level (mmol/L)	1.82 (1.54–2.23)	1.94 (1.53–2.49)	0.336	1.85 (1.49–2.27)	2.49 (1.94–2.95)	0.001*
Calcium-phosphorus product level (mg^2^/dL^2^)	51.42 (40.83–66.64)	57.72 (44.09–74.32)	0.144	52.15 (40.02–67.16)	75.00 (58.43–92.14)	0.001*
Homocysteine level (μmol/L)	18.4 (15.1–24.1)	20.1 (15.8–26.4)	0.692	19.9 (14.8–25.6)	20.7 (16.6–26.7)	0.162

BP: blood pressure; HbA1C: glycated hemoglobin A1C; hs-CRP: high-sensitivity C-reactive protein; BUN: blood urea nitrogen; iPTH: intact parathyroid hormone; LDL-C: low-density lipoprotein cholesterol; RAC: radial artery calcification.

*p < 0.05.

### Correlations between radial artery calcification and diabetes mellitus

The left side of Table 4 shows the differences in clinical and laboratory results between patients with and without diabetes mellitus. Patients with diabetes mellitus had a higher prevalence of RAC (41.9% vs. 10.2%; *p* < 0.01), older age (median, 61.5 vs. 48.0 years; *p* < 0.01), higher systolic BP (median, 165.0 vs. 155.0 mm Hg; *p* < 0.01), higher triglyceride level (median, 1.61 vs. 1.24 mg/L; *p* < 0.05), higher HbA1c level (median, 6.5 vs. 5.5 mmol/L; *p* < 0.01), and less frequent dialysis duration >5 years (4% vs. 28%; *p* < 0.01), lower diastolic BP (median, 83.5 vs. 95.5 mm Hg; *p* < 0.01), and lower albumin (median, 31.5 vs. 34.4 g/L; *p* < 0.01) and phosphate levels (median, 1.77 vs. 2.06 mmol/L; *p* < 0.05). The right side of Table 4 compares the clinical and laboratory results between patients with diabetes mellitus ≥15 years and those with diabetes mellitus <15 years. If patients had diabetes mellitus ≥15 years, there was a higher prevalence of RAC (59.1% vs. 17.6%; *p* < 0.05) and older age (median, 67.5 vs. 58.0 years; *p* < 0.01).

**Table 4.** Correlations between diabetes mellitus and clinical and laboratory variables.

Variable	Non-diabetes mellitus (*n* = 118)	Diabetes mellitus (*n* = 62)	*p*	Diabetes mellitu*s* < 15 years (*n* = 40)	Diabetes mellitu*s* ≥ 15 years (*n* = 22)	*p*
Male sex (%)	69 (58.5)	39 (62.9)	0.564	25 (62.5)	14 (63.6)	0.929
Smoking habit (%)	25 (21.2)	14 (22.6)	0.829	8 (20.0)	6 (27.3)	0.512
Dialysis duratio*n* > 5 years (%)	28 (23.7)	4 (6.5)	0.004*	1 (2.5)	3 (13.6)	0.090
RAC (%)	12 (10.2)	25 (41.9)	0.001*	13 (32.5)	13 (59.1)	0.042*
Age (years)	48.0 (37.0–58.0)	61.5 (47.8–69.3)	0.001*	58.0 (46.0–66.8)	67.5 (51.0–72.5)	0.007*
Systolic BP (mm Hg)	155.0 (138.0–170.0)	165.0 (154.8–173.0)	0.002*	163.5 (154.5–172.8)	168.0 (154.3–180.8)	0.648
Diastolic BP (mm Hg)	95.5 (81.8–103.0)	83.5 (74.8–92.3)	0.001*	85.0 (76.0–93.0)	80.5 (72.0–89.8)	0.210
Hemoglobin level (g/L)	98.0 (81.0–114.3)	96.0 (81.5–112.5)	0.552	91.5 (80.0–102.8)	100.0 (89.0–122.3)	0.079
Triglyceride level (mmol/L)	1.24 (0.94–1.70)	1.61 (1.02–2.12)	0.043*	1.61 (0.98–2.16)	1.69 (1.07–2.14)	0.545
Cholesterol level (mmol/L)	4.29 (3.71–4.93)	4.19 (3.59–4.85)	0.817	4.37 (3.71–5.18)	3.80 (3.23–4.66)	0.246
LDL-cholesterol level (mmol/L)	2.32 (1.90–2.80)	2.20 (1.80–2.92)	0.873	2.20 (1.90–3.20)	2.10 (1.55–2.45)	0.063
Creatinine level (μmol/L)	942.0 (634.3–1267.5)	767.0 (579.5–874.5)	0.873	770.0 (578.5–978.8)	758.0 (646.8–827.0)	0.958
BUN level (mmol/L)	21.2 (16.5–27.1)	19.5 (14.5–24.2)	0.128	19.9 (14.0–24.5)	18.6 (14.5–23.1)	0.837
Albumin level (g/L)	34.4 (31.0–37.6)	31.5 (27.5–35.4)	0.001*	31.0 (24.2–34.8)	33.0 (30.4–35.9)	0.134
hs-CRP level (mg/L)	4.0 (2.0–7.3)	3.0 (1.0–6.0)	0.239	3.0 (1.0–5.0)	4.0 (1.0–7.5)	0.534
HbA1c level (%)	5.5 (4.9–5.8)	6.5 (5.9–7.8)	0.001*	6.3 (5.9–7.6)	7.0 (6.1–8.3)	0.275
Calcium carbonate supplements (%)	100 (84.7)	48 (77.4)	0.222	31 (77.5)	17 (77.3)	0.984
Calcitriol supplements (%)	110 (93.2)	53 (85.5)	0.092	35 (87.5)	18 (81.8)	0.547
iPTH level (pg/mL)	246.3 (150.0–542.9)	200.0 (120.0–382.5)	0.051	200.0 (119.8–328.4)	249.7 (122.5–476.2)	0.696
Calcium level (mmol/L)	2.40 (2.22–2.50)	2.42 (2.21–2.56)	0.423	2.42 (2.21–2.54)	2.45 (2.23–2.59)	0.636
Phosphate level (mmol/L)	2.06 (1.59–2.51)	1.77 (1.45–2.25)	0.047*	1.73 (1.39–2.43)	1.79 (1.53–1.97)	0.780
Calcium-phosphorus product level (mg^2^/dL^2^)	59.08 (44.75–74.46)	50.83 (41.21–65.95)	0.088	50.83 (39.90–67.81)	50.32 (42.50–64.57)	0.664
Homocysteine level (μmol/L)	20.3 (16.2–26.4)	19.3 (14.3–24.6)	0.116	18.8 (13.7–24.1)	20.3 (14.2–26.8)	0.730

BP: blood pressure; HbA1C: hemoglobin A1C; hs-CRP: high-sensitivity C-reactive protein; BUN: blood urea nitrogen; iPTH: intact parathyroid hormone; LDL-C: low-density lipoprotein cholesterol; RAC: radial artery calcification.

*p < 0.05.

### Correlations of radial artery calcification level with various parameters

A significant correlation was observed between radial artery calcification level and related parameters (Table 5). radial artery calcification level showed a significantly positive correlation with dialysis duration (*r* = 0.186; *p* < 0.05), diabetes mellitus duration (*r* = 0.186; *p* < 0.01), HbA1c level (*r* = 0.222, *p* < 0.01) and Calcium level (*r* = 0.224, *p* < 0.01). No significant correlations were observed between radial artery calcification level and age, smoking habit, triglyceride level, LDL-cholesterol level, iPTH level, phosphate level and calcium-phosphorus product level respectively.

**Table 5.** Correlations of radial artery calcification level with various parameters.

Variable	radial artery calcification level	
	*r*	*p*
Dialysis duration (months)	0.186	0.012*
Diabetes mellitus duration (months)	0.393	0.001*
HbA1c level (%)	0.222	0.003*
Calcium level (mmol/L)	0.224	0.002*
Albumin level (g/L)	-.0101	0.179
Age (years)	0.016	0.834
Smoking habit (%)	0.014	0.850
Triglyceride level (mmol/L)	0.032	0.668
Cholesterol level (mmol/L)	−0.029	0.703
LDL-cholesterol level (mmol/L)	−0.008	0.919
Homocysteine level (μmol/L)	−0.085	0.258
Calcium carbonate supplements (%)	0.072	0.338
Calcitriol supplements (%)	0.027	0.720
iPTH level (pg/mL)	0.019	0.790
Phosphate level (mmol/L)	−0.012	0.868
Calcium-phosphorus product level (mg^2^/dL^2^)	0.057	0.449
Systolic BP (mm Hg)	0.099	0.186
Diastolic BP (mm Hg)	−0.107	0.151
Male sex (%)	0.063	0.399
Hemoglobin level (g/L)	−0.032	0.672
Creatinine level (μmol/L)	−0.036	0.635
BUN level (mmol/L)	−0.014	0.857
hs-CRP level (mg/L)	0.048	0.524

VC: vascular calcification; BP: blood pressure; HbA1C: glycated hemoglobin A1C; hs-CRP: high-sensitivity C-reactive protein; BUN: blood urea nitrogen; iPTH: intact parathyroid hormone; LDL-C: low-density lipoprotein cholesterol.

## Discussion

The histological examination of the obtained radial artery walls enabled us to evaluate not only the calcification degrees but also their locations. AIC is associated with inflammation and the advancement of atherosclerosis, while adjacent regions of the vessel wall may remain remarkably normal. AMC develops independently of atherosclerosis and is characterized by diffuse mineral deposition throughout the vascular tree [3,8]. In the present study, we found mineral deposits in 21.1% of the examined radial arteries using alizarin red S, which is more sensitive than von Kossa staining. Basophilic mineral deposits were seen almost entirely in the media layer of the radial arteries, which presented different degrees of advancement. The arterial specimens stained with alizarin red S did not demonstrate intimal layer calcification. Our histological findings confirm the results of other studies [9,10].

VC is the result of multiple factors acting together over a certain period, and the transdifferentiation of VSMCs into osteoblast-like cells is the central part of the production of VC [11]. Consistent with our previous studies, RAC was confirmed by tissue biopsy after expanding the enrolled patients, and then its relationship with traditional risk factors of VC and the risk factors characteristic of CKD were analyzed. It was found that RAC was still associated with dialysis duration >5 years and diabetes. Other traditional vascular risk factor – including LDL and triglycerides are not so important. Further logistic regression analysis found that after correcting for the interference factors, dialysis duration >5 years and diabetes are independent risk factors for the development of RAC in patients with ESKD. The characteristics of RAC in patients on dialysis and non-dialysis, duration of HD, diabetes and non-diabetes, and medical history of diabetes were further analyzed in the following subgroups.

HD is an important treatment for patients with ESKD. In our subgroup analysis, we found that HD could improve the anemia status of patients with ESKD, reduce the level of blood urea nitrogen, and improve he blood calcium, but compared with conservative treatment of patients with ESKD, it also causes an increase in systemic inflammation. The increase in blood calcium leads to an increase in the deposition of calcium and phosphorus, which accumulates in VSMCs passively and finally results in the calcification of VSMC [12]. However, the state of micro-inflammation of blood vessels can occur in the early stage of CKD and lead to the genesis of VC through various pathways [13], one of which is by disrupting the LDL receptor. We found no significant difference in RAC between patients treated with HD and those who were under conservative treatment, which suggests that simply starting HD is not the primary cause of RAC.

With the extension of HD time, more factors will cause VC involvement. Therefore, we referred to the literature, compared the results of our previous studies, and used dialysis duration >5 years as a cutoff point for long-term dialysis. In the subgroup analysis of HD time, we found that when dialysis duration was >5 years, the levels of corrected blood calcium, blood phosphorus, calcium-phosphorus product, parathyroid hormone, and calcium supplement were significantly higher than those of HD time <5 years, which demonstrated the previous relevant opinions about VC caused by ESKD [14–16]. From a histopathological viewpoint, we found a significant difference in the incidence of RAC between two groups, which suggested that HD requires time for accumulation to lead to histologically detectable calcification, and VC detected by imaging showed similar time-related results between VC and HD [17]. Under the influence of secondary hyperparathyroidism, calcium and phosphorus metabolism disorders, increased calcium and phosphorus loads (food and iatrogenic drugs), HD cannulation, and long-term stimulation of blood turbulence on the vascular wall, etc. [18]. Patients with ESKD will eventually have histopathologically detectable RAC after a certain period of accumulation.

Diabetes mellitus is the major cause of ESKD and one of the major risk factors of cardiovascular disease [19]. Among the included patients with ESKD, 34.4% had diabetic mellitus. In the subgroup analysis, we compared diabetic and non-diabetic patients with ESKD. We found that in the diabetes mellitus group, although the dialysis duration and blood phosphorus were lower than those in the non-diabetes mellitus group, the RAC incidence was much higher than that in the non-diabetes mellitus group, suggesting that diabetes mellitus itself is a strong VC-causing factor. Current studies have found that VC caused by diabetes mellitus may be related to inflammatory status, oxidative stress-level bone mineral metabolism disorders, and other factors [20]. Patients in the diabetes mellitus group were also older and had a higher pulse pressure difference (systolic blood pressure was less than the diastolic blood pressure) and triglyceride level. Older age and disorders of blood lipid metabolism can lead to vascular stiffness or calcification [21]. The high blood vessel pulse pressure will definifitely increase, and blood pressure will be more difficult to control. Moreover, ESKD patients with diabetes mellitus have a more severe degree of VC than non-diabetic patients and in addition to the risk factors for CKD, they are also associated with diabetes-specific endothelial dysfunction, hyperlipidemia, hyperinsulinemia, poor glycemic control, and tissue deficiency. Factors such as oxygen are related too [22]. Finally, the proportion of HD patients with diabetes mellitus >5 years was significantly less than that in non-diabetic patients. We consider that this is related to the general age of patients with diabetes mellitus and that a more severe degree of VC increases the risk of death due to cardiovascular disease.

In order to explore whether the course of diabetes mellitus also has a further aggravating effect on VC in patients with ESKD, we performed a further subgroup analysis of ESKD patients with diabetes mellitus. We found that significant differences in RAC can occur when the patients’ history of diabetes is >15 years and when they are older. The conclusions are similar to those regarding the coronary arteries. The duration of diabetes is related to the calcification index of the coronary arteries [23]. VC is regarded as a decline of age-related minerals and the extracellular matrix passively deposited in blood vessels [24]. The hydroxyapatite deposition in blood vessels has a time course, reflecting the differences between the diabetes course and age. Moreover, as shown in Table 3, we discovered that if dialysis duration is >5 years, there were higher levels of hemoglobin and albumin. This is because the patient's residual kidney function is almost lost. The protein excreted from the urine is reduced, and the hemoglobin level is gradually increased. At the same time, the standardized use of iron and erythropoietin causes an increase in the hemoglobin level.

Finally, we analyzed the correlation between radial artery calcification level and related parameters. We found that dialysis duration and the course of diabetes mellitus were positively correlated with the degree of vascular calcification, which was similar to the results we have discussed above. The degree of vascular calcification was also correlated with the accumulation of time. Under the long-term effect of factors leading to vascular calcification, the degree of vascular calcification could get aggravated. The degree of radial artery calcification is also positively correlated with HbA1c level and calcium level. With worsening regulation of blood glucose level and increasing level of calcium, the degree of vascular calcification gets aggravated.

There are some limitations to this study. First, the cross-sectional nature precludes the determination of cause and effect. Moreover, the arterial specimens obtained during surgery are imperfect in terms of the actual calcification in the entire artery during AVF surgery. Furthermore, there was a lack of ultrasonography and radiology verification of the degree of calcification in the vessels. Finally, a quantitative scoring method was not used to assess calcification.

## Conclusions

Small pieces of the radial arteries obtained during the creation of AVF for HD may successfully be the material source for VC histological assessment. In patients with ESKD, dialysis duration >5 years and diabetes mellitus predict RAC. These results indicate that a combination of long-time dialysis and hyperglycemic conditions exerts a synergistic effect on RAC.
